# A New Understanding of Long Non-Coding RNA in Hepatocellular Carcinoma—From m^6^A Modification to Blood Biomarkers

**DOI:** 10.3390/cells12182272

**Published:** 2023-09-14

**Authors:** Jung Woo Eun, Jae Youn Cheong, Jee-Yeong Jeong, Hyung Seok Kim

**Affiliations:** 1 Department of Gastroenterology, Ajou University School of Medicine, 164 World cup-ro, Yeongtong-gu, Suwon 16499, Republic of Korea; jetaimebin@gmail.com (J.W.E.); jaeyoun620@gmail.com (J.Y.C.); 2 Department of Biochemistry, College of Medicine, Kosin University, Seo-gu, Busan 49267, Republic of Korea; jyjeong@kosin.ac.kr; 3 Institute for Medical Science, College of Medicine, Kosin University, Seo-gu, Busan 49267, Republic of Korea

**Keywords:** hepatocellular carcinoma, long non-coding RNA, tumor microenvironment, N^6^-methyladenosine, biomarker

## Abstract

With recent advancements in biological research, long non-coding RNAs (lncRNAs) with lengths exceeding 200 nucleotides have emerged as pivotal regulators of gene expression and cellular phenotypic modulation. Despite initial skepticism due to their low sequence conservation and expression levels, their significance in various biological processes has become increasingly apparent. We provided an overview of lncRNAs and discussed their defining features and modes of operation. We then explored their crucial function in the hepatocarcinogenesis process, elucidating their complex involvement in hepatocellular carcinoma (HCC). The influential role of lncRNAs within the HCC tumor microenvironment is emphasized, illustrating their potential as key modulators of disease dynamics. We also investigated the significant influence of N^6^-methyladenosine (m^6^A) modification on lncRNA function in HCC, enhancing our understanding of both their roles and their upstream regulators. Additionally, the potential of lncRNAs as promising biomarkers was discussed in liver cancer diagnosis, suggesting a novel avenue for future research and clinical application. Finally, our work underscored the dual potential of lncRNAs as both contributors to HCC pathogenesis and innovative tools for its diagnosis. Existing challenges and prospective trajectories in lncRNA research are also discussed, emphasizing their potential in advancing liver cancer research.

## 1. Introduction

In recent decades, advancements in biological technology have profoundly evolved our comprehension of genomic information. This shift was largely attributed to the discovery of non-coding RNAs (ncRNAs), which, while not encoding proteins, regulate various biological processes [1]. The categories of ncRNAs—microRNAs (miRNAs), circular RNAs (circRNAs), PIWI-interacting RNAs (piRNAs), and small nucleolar RNAs (snoRNAs)—each have unique roles, such as gene expression regulation, miRNA sponging, genome stability maintenance, and guiding chemical RNA modifications, respectively [2,3].

Among these ncRNAs, long non-coding RNAs (lncRNAs)—transcripts longer than 200 nucleotides—have emerged as crucial regulators in determining cellular fate. They serve as critical molecular players in diverse biological processes, including X-chromosome inactivation and genomic imprinting [4]. These lncRNAs were initially considered as non-functional transcripts due to their generally low sequence conservation and expression levels. However, the increasing number of publications highlighting the dynamic expression and biological functions of lncRNAs, together with the advent of novel technologies facilitating their identification and characterization, have redefined our understanding of this perception [5]. Moreover, their aberrant expression and function have been implicated in various cancers, particularly hepatocellular carcinoma (HCC) [6].

This review explored the recent advances in research on the complex roles and mechanisms of lncRNA dysregulation in HCC. We provided a comprehensive exploration of their implications in disease pathogenesis and potential as diagnostic markers. Additionally, we included information specifically on the N^6^-methyladenosine (m^6^A) modification, offering a more detailed understanding of its influence. Luo et al. concluded a critical facet of lncRNA biology is the m^6^A modification; it represents the most predominant internal modification observed in eukaryotic RNAs, including lncRNAs, exerting a pivotal role in RNA metabolism and functionality [7]. The modulation of lncRNA structures and functions by m^6^A introduces additional layers of regulatory intricacy [7,8]. Importantly, Sivasudhan et al. highlighted in their review the significance of lncRNA alterations via m^6^A modifications, emphasizing their potential influence on disease progression and therapeutic outcomes in HCC [9]. Finally, we identified current challenges and discussed potential future research directions in this compelling field. Despite the uncertainty and controversy that have involved lncRNA research, these molecules play pivotal roles in regulating cellular functions, with significant implications for HCC, highlighting their significance in biomedical research.

## 2. Understanding lncRNA: Its Definition and Mechanism of Action

LncRNAs play a pivotal role in the intricate regulation of gene expression. As previously reviewed, lncRNAs’ influence spans multiple levels of gene regulation, from reshaping chromatin structures to guiding post-transcriptional modifications [10,11]. A fundamental mechanism underlying their function is their ability to interact with various cellular components, such as DNA, RNA, and proteins [12]. Through these interactions, lncRNAs become essential modulators of both cellular architecture and activity.

### 2.1. Signal

**Figure 1:**
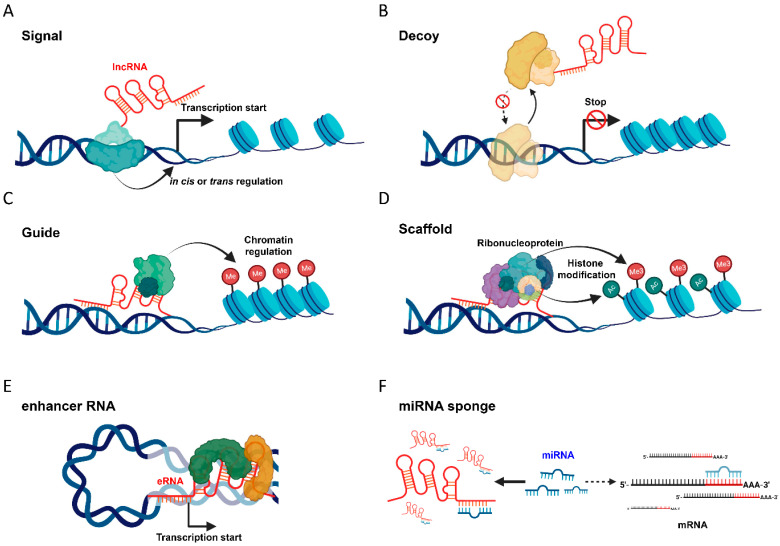
Diverse mechanisms of lncRNAs’ functions in cellular regulation. (**A**) Signal. LncRNAs act as molecular indicators, responding to various cellular stimuli. (**B**) Decoy. LncRNAs can bind and sequester transcription factors or other proteins, preventing them from interacting with their target genomic loci. (**C**) Guide. LncRNAs direct chromatin-modifying enzymes to specific genomic regions, enabling targeted epigenetic modifications. (**D**) Scaffold. LncRNAs facilitate the formation of multi-protein complexes, providing a structural platform for these assemblies. (**E**) Enhancer RNA. LncRNAs can function as enhancers, looping DNA to bring distant regions into proximity for transcriptional activation. (**F**) miRNA Sponge. LncRNAs can act as sponges for miRNAs, sequestering them and preventing them from binding to their target mRNAs, thus inhibiting miRNA-mediated gene repression.

One of the primary modes of lncRNA function is acting as signals (Figure 1A). These molecular indicators are transcribed in response to diverse cellular stimuli, serving as indicators of specific cellular states or events. Their transcription often reflects changes in the cellular environment, such as stress, differentiation, or developmental cues. For instance, the lncRNA HOTAIR is transcribed in response to certain oncogenic signals and plays a pivotal role in regulating gene expression patterns associated with cancer progression [13]. Similarly, XIST is transcribed during early female embryonic development, signaling the initiation of X-chromosome inactivation [4]. These lncRNAs, by acting as signals, provide the cell with a dynamic mechanism to rapidly respond to internal or external changes, ensuring appropriate cellular reactions and adaptations. Their signaling role underscores the complexity and versatility of the non-coding genome in cellular regulation and function.

### 2.2. Decoy

Another well-known function of lncRNAs is acting as decoys (Figure 1B). In this capacity, lncRNAs can bind to transcription factors or other proteins, effectively sequestering them away from their target genomic loci [14,15]. For instance, the lncRNA Growth Arrest-Specifc 5 (GAS5) serves as a decoy by binding to the glucocorticoid receptor (GR). Under conditions of growth arrest, GAS5 accumulates and binds to the DNA-binding domain of the GR. This prevents the GR from binding to its glucocorticoid response elements (GREs) on DNA, thereby inhibiting the transcription of glucocorticoid-responsive genes. As a result, GAS5 plays a crucial role in modulating cell growth and the cellular response to stress through its decoy function [16]. These lncRNA–protein interactions are responsive to cellular conditions, with factors such as environmental stress potentially affecting lncRNA expression levels and, in turn, their decoy function. 

### 2.3. Guide

LncRNAs can also act as guides, directing the localization of chromatin-modifying enzymes to specific genomic regions (Figure 1C). This targeted recruitment enables precise epigenetic modifications, which can subsequently lead to changes in gene expression profiles. As an example, a recent study demonstrated that TARID (TCF21 antisense RNA inducing demethylation) functioned by partnering with growth arrest and DNA-damage-inducible alpha protein (GADD45A). This partnership steered the DNA demethylation machinery to specific gene loci in cancer cells, thereby regulating gene expression [17].

### 2.4. Scaffold

In their function as scaffolds, lncRNAs can facilitate the formation of multi-protein complexes, providing a structural platform for the assembly of these complexes. This function aids in the organization and coordination of various cellular processes, including signal transduction and the regulation of gene expression. For instance, the RNA-binding NONO-PSF heterodimer and NEAT1 are both involved in enhancing the processing of primary miRNAs (pri-miRNAs) in HeLa cells. They interact with each other and other RNA-binding proteins, facilitating the microprocessor’s access to pri-miRNAs. This suggests that lncRNA may play a central role in regulating small noncoding RNAs in the nucleus [18].

### 2.5. Enhancer RNA

LncRNAs can function as enhancers, augmenting the transcription of nearby genes by looping the DNA, bringing distant regions into close proximity for transcriptional activation (Figure 1E). They can modulate transcription either *in cis*, regulating neighboring genes on the same chromosome, or *in trans*, influencing genes on different chromosomes [19]. Miao et al. identified the role of LEENE (a lncRNA that enhances eNOS expression) in enhancing the expression of endothelial nitric oxide synthase (eNOS), a key factor in vascular function. LEENE facilitated RNA Pol II’s binding to the *eNOS* promoter, positively modulating the synthesis of *eNOS* mRNA and promoting endothelial function [20].

Beyond their known roles, lncRNAs possess a plethora of unexplored functions, validating the perspective that they might perform a limitless array of tasks within a biological context. Not only are lncRNAs pivotal for cellular differentiation and development, but they also influence an extensive range of physiological processes. These encompass DNA damage response, immune system regulation, metabolic processes, and synapse function [21,22,23,24]. The initial skepticism about their importance, due to their lack of protein-coding potential, has been substantially overcome by a growing body of research. This shift in perspective underlines not only the importance of understanding the full range of lncRNA functions, but also their potential implications in health and disease.

### 2.6. MiRNA Sponge

One of the prominent functions of lncRNAs is their ability to act as miRNA sponges. By binding to miRNAs, these lncRNAs effectively inhibit the miRNAs from associating with their target mRNAs, thereby modulating post-transcriptional regulation (Figure 1F).

A classic example is the lncRNA HOTAIR, which has been reported to sponge miR-34a, a tumor suppressor miRNA. By sequestering miR-34a, HOTAIR can promote oncogenic pathways in certain cancer types, pointing to the significance of lncRNA–miRNA interactions in disease progression [25,26,27]. Furthermore, lncRNA–miRNA interactions introduce an added layer of post-transcriptional regulation complexity. These interactions not only modulate individual gene expressions, but also impact broader cellular pathways and processes. Such processes include cell proliferation, differentiation, and apoptosis, where the balance between lncRNAs and miRNAs plays a crucial role [28,29,30].

## 3. Pivotal Role of lncRNA in Hepatocarcinogenesis

Due to the increasing demand for better diagnostic and therapeutic approaches, the molecular underpinnings of HCC have become a focal point of research. As Ghafouri-Fard et al. and Abbastabar et al. concluded in their reviews, multiple lncRNAs have been shown to either promote or inhibit HCC progression, affecting processes such as cell proliferation, migration, and invasion [31,32]. The following lncRNAs exemplify these characteristics in the context of HCC.

### 3.1. HOTAIR (HOX Transcript Antisense RNA)

HOTAIR is an oncogenic lncRNA found to be upregulated in HCC. It originates from the antisense strand of the Homeobox (HOX) gene cluster and plays a significant role in cancer progression [33]. HOTAIR was reported to play a crucial role in HCC by regulating cell growth, migration, invasion, and apoptosis. Its overexpression was often associated with greater tumor size, metastasis, and poor prognosis [34,35,36].

Specifically, HOTAIR plays a key role through its interactions with Polycomb Repressive Complex 2 (PRC2) and Lysine Specific Demethylase 1 (LSD1), which are crucial players in gene silencing [37]. More specifically, HOTAIR acts as a scaffold, binding to EZH2, a subunit of PRC2, thereby facilitating PRC2’s role in repressing gene transcription through the trimethylation of histone H3 at lysine 27 (H3K27me3) [38]. At the same time, HOTAIR can bind to LSD1, which in cooperation with CoREST/REST forms a multi-protein complex involved in gene silencing. This interaction allows HOTAIR to contribute to the silencing of miRNA through its association with PRC2 and LSD1 [39]. Furthermore, HOTAIR is implicated in the regulation of SUZ12, a key binding subunit of PRC2. Overexpression of HOTAIR accelerates the proteasome degradation of SUZ12 and enhances the ubiquitination of SUZ12, facilitated by PLK1. Another interaction of HOTAIR is with the DEAD-box helicase protein 5 (DDX5). This interaction stabilizes SUZ12, reinforcing SUZ12- and PRC2-mediated gene silencing. DDX5 replaces the original Mex-3 RNA binding family member B (Mex3b) linked to HOTAIR, thereby stabilizing the HOTAIR–PRC2 interaction [40]. In HCC patients, overexpression of HOTAIR and PLK1, more than twice the normal levels, was associated with a significant increase in the expression of PRC2 target genes and EPCAM, underscoring the impact of HOTAIR on the epigenetic regulation in HCC [39].

Moreover, HOTAIR also functions as a molecular sponge, sequestering miRNAs like miR-218, which suppresses tumorigenesis. By sponging miRNAs, HOTAIR prevented their anti-cancer effects, leading to enhanced cell proliferation and metastasis [34].

### 3.2. NEAT1 (Nuclear Enriched Abundant Transcript 1)

NEAT1 is a central component of paraspeckles, specialized sub-nuclear bodies, and plays a pivotal role in their formation and integrity [41]. Over the years, research has shown that NEAT1 is aberrantly upregulated in a variety of cancers. This heightened expression often correlates with a poorer prognosis for patients, making it a potential biomarker for disease progression [42,43,44]. In the context of liver diseases, NEAT1’s role is multifaceted. It has been implicated in accelerating the progression of non-alcoholic fatty liver disease (NAFLD), liver fibrosis, and HCC. However, in conditions characterized by an acute deterioration of liver function in patients with pre-existing chronic liver disease, NEAT1 assumes a protective role by mitigating the inflammatory response [45].

Additional studies in HCC confirmed that NEAT1 is typically overexpressed, promoting cell proliferation, migration, and invasion [46]. Mechanistically, NEAT1 forms a complex with U2AF65, which in turn boosts the expression of hnRNP A2, a known driver in HCC [47]. Another layer of regulation involves HIF-2α, which enhances NEAT1 expression, subsequently influencing the epithelial-mesenchymal transition, a critical process in cancer metastasis [48]. A significant aspect of NEAT1’s function is its ability to act as a molecular sponge for a range of miRNAs. By sequestering these miRNAs, NEAT1 restores the expression of specific genes that these miRNAs would otherwise inhibit [49,50,51]. 

Apart from its role as a miRNA sponge, NEAT1 also plays a pivotal role in ferroptosis, a unique form of cell death driven by iron-dependent lipid peroxidation, crucial for tumor development and drug resistance [52]. Recent studies have shown that two ferroptosis inducers, erastin and RSL3, elevate NEAT1 expression by enhancing p53’s binding to the NEAT1 promoter. Once upregulated, NEAT1 boosts MIOX expression by competitively binding to miR-362-3p. This leads to an increase in ROS production and a decrease in intracellular NADPH and GSH levels, amplifying the effects of erastin and RSL3. Notably, overexpressing NEAT1 enhances the anti-tumor effects by intensifying ferroptosis in vitro and in vivo [51]. 

Additionally, NEAT1’s role extends to influencing drug resistance in HCC. It had been shown to synergistically enhance cisplatin resistance in certain liver cancer cells [53]. Furthermore, its involvement in the resistance to sorafenib, a primary therapeutic agent for HCC, has been shown. To be specific, inhibition of NEAT1 amplifies the efficacy of sorafenib, resulting in increased drug-induced cell death and notably smaller tumors in nude mice than with just sorafenib treatment [54].

### 3.3. HULC (Highly Upregulated in Liver Cancer)

In liver cancer, an increase in the lncRNA HULC, driven by the protein CREB, significantly influences cellular mechanisms by altering YB-1 phosphorylation patterns, which is key in hepatocarcinogenesis [55,56]. HULC also blocks the programmed cell death, or apoptosis, in these cancer cells, specifically when triggered by miR-9 [57]. Another protein, Hepatitis B virus X (HBX), also induces increased levels of HULC and reduces p18, which in turn helps HCC to grow [58]. Given these roles, the overexpression of HULC suggests its potential as a noninvasive biomarker for diagnosis and prognosis [59]. This previous research highlighted the role of HULC in the growth and progression of HCC cells.

### 3.4. MALAT1 (Metastasis Associated Lung Adenocarcinoma Transcript 1)

MALAT1 is a recognized oncogenic lncRNA that plays a crucial role in the progression of HCC. This lncRNA functions through various pathways, particularly serving as a molecular sponge, and has been observed to be overexpressed in HCC [60]. To be specific, the function of MALAT1 is its ability to bind and sequester various miRNAs, thereby influencing their target function. For instance, MALAT1 reduces the expression of miR-204, leading to an increase in SIRT1 levels and the facilitation of the epithelial–mesenchymal transition (EMT) [61]. Additionally, MALAT1 sequesters miR-143-3p, leading to the upregulation of FGF1- and EMT-promoting proteins [62]. Furthermore, MALAT1’s interaction with miR-200a leads to increased levels of proteins involved in EMT and cell proliferation [63]. Moreover, MALAT1’s interaction with miRNAs such as miR-124-3p and miR-195 results in an upregulation of cell proliferation and invasion facilitating proteins, further highlighting its role in HCC progression [64,65]. Lastly, through downregulation of miR-22, MALAT1 promotes EMT and recruits EZH2 to suppress E-cadherin and miR-22 expression [66]. 

Beyond the aforementioned lncRNAs, a vast number of additional lncRNAs have been recently discovered and their functions are deeply intertwined with the onset and progression of HCC (Table 1). This has solidified their position as a distinct subject of study within the field of oncology.

## 4. LncRNAs as Key Modulators of the HCC Tumor Microenvironment

The evolving landscape of cancer biology has recognized the pivotal role of lncRNAs in modulating the tumor microenvironment (TME). Park et al. reviewed that these lncRNAs, by orchestrating intricate interactions within the TME, contribute to various aspects of cancer progression including uncontrolled growth, metastasis, and immune evasion [100].

The immune landscape within HCC is a complex interplay of multiple cell types, influenced and orchestrated by lncRNAs. Jiang et al. demonstrated that the polarization of macrophages, a crucial cellular component in the environment of HCC, is indicative of this interplay [101]. 

To be specific, the overexpression of MALAT1 in HCC cells promotes angiogenesis and fosters an immunosuppressive environment. This occurs through MALAT1’s interaction with miR-140, inhibiting miR-140’s activity, and consequently increasing VEGF-A production which aids HCC progression by promoting angiogenesis and favoring the polarization of macrophages towards the M2 immunosuppressive subset [102].

Beyond macrophages, lncRNAs also play significant roles in modulating T-cell functions within HCC, impacting the disease’s progression and immune escape mechanisms. For example, lncRNA epidermal growth factor receptor (lnc-EGFR) is highly expressed in regulatory T cells (Tregs) in HCC. Lnc-EGFR interacts with EGFR, inhibits its ubiquitination by c-CBL, and amplifies downstream signaling via AP-1/NFAT1, promoting Treg-cell differentiation and immune evasion [103].

On the contrary, the lncRNA fetal-lethal non-coding developmental regulatory RNA (FENDRR) acts as a sponge for miR-423-5p, impeding Tregs’ immune-suppressive activities. Overexpressed FENDRR competitively binds miR-423-5p and upregulates growth arrest and DNA-damage-inducible beta protein (GADD45B), which inversely correlate with Treg-cell number, thereby reducing immunosuppressive cytokines TGF-β and IL-10 and inducing tumor-cell apoptosis [96]. Moreover, lncRNAs Tims and lncNNT-AS1 are associated with reduced infiltration of tumor CD4 and CD8 T cells, influencing clinical outcomes and responses to immunotherapies [104].

Another lncRNA, Myocardial Infarction Associated Transcript (MIAT), shows elevated expression in various cells implicated in the disease, including tumor cells themselves: FoxP3+ Tregs, PD-1+ CD8+ T cells, and GZMK+ CD8+ T cells. Furthermore, the upregulation of MIAT is associated with how well patients respond to sorafenib. This lncRNA’s expression also has a significant correlation with the presence of PD-L1, a protein involved in immune evasion by cancer cells [105].

## 5. Implications of m^6^A Modification on lncRNA in HCC 

**Figure 2:**
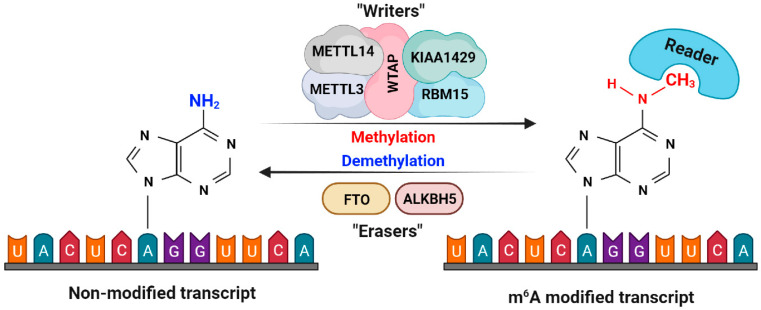
A schematic view of m^6^A modification machinery. The m^6^A methyltransferase complex, composed of METTL3, METTL14, KIAA1429, RBM15, and WTAP, is responsible for adding the m^6^A modification. In contrast, demethylases, including FTO and ALKBH5, are depicted removing the m^6^A marks. This m^6^A modification plays a pivotal role in determining cellular fate and is involved in the onset of diseases such as cancer.

N^6^-methyladenosine (m^6^A) is the most prevalent internal modification in eukaryotic RNA. It plays critical roles in various biological processes, including mRNA splicing, export, stability, and translation efficiency [106,107,108]. The addition of the m^6^A modification is catalyzed by an enzyme complex known as the m^6^A methyltransferase complex, including METTL3, METTL14, KIAA1429, RBM15, and WTAP [109,110,111,112]. On the other hand, demethylases, such as FTO and ALKBH5, remove m^6^A modifications [99,113,114]. Proteins that recognize m^6^A modifications, often referred to as reader proteins, such as YTH domain-containing proteins, recognize these modifications and influence the fate of m^6^A-modified RNAs [115,116,117]. Figure 2 depicts the mode of action for the m^6^A modification mechanism of this protein complex.

Lately, many studies have demonstrated the intricate roles of m^6^A modification on lncRNAs in HCC progression. One such example is LINC00958, a lncRNA found to be overexpressed in HCC. LINC00958 acted as a molecular sponge for miR-3619-5p, leading to the upregulation of hepatoma-derived growth factor (HDGF), thereby promoting HCC progression. Its overexpression was facilitated by the m^6^A methyltransferase METTL3, suggesting a critical interplay between m^6^A modification and lncRNA functionality [118].

An m^6^A-related lncRNA prognostic signature involving LINC02362, SNHG20, and SNHG6 was identified as a powerful predictor of patient survival, reflecting the close connection between the m^6^A modification landscape, lncRNA dynamics, and patient outcomes [119]. Additionally, MEG3 demonstrated tumor-suppressive roles through the miR-544b/BTG2 signaling pathway upon upregulation by m^6^A modification [94].

In a related study, ALKBH5-mediated m^6^A demethylation downregulated LINC02551, a crucial lncRNA for HCC growth and metastasis, indicating the importance of the balance between methylation and demethylation processes in the m^6^A–lncRNA axis [86]. Further investigations revealed the promoting role of METTL16 in HCC progression through the downregulation of the tumor suppressor RAB11B-AS1 via an m^6^A–YTHDF2-dependent mechanism [99].

The role of m^6^A modification was also underscored in immune evasion, with lipopolysaccharide (LPS) found to increase PD-L1 expression through the m^6^A modification of MIR155HG, a process essential for HCC immune evasion [120]. The lncRNA ARHGAP5-AS1, which exhibited elevated m^6^A levels on its transcript, is overexpressed in HCC. It was modulated by METTL14, which functions as its m^6^A writer, and by IGF2BP2, which acts as the m^6^A reader. Interestingly, the oncogenic ARHGAP5-AS1 diminished the interactions between CSDE1 and TRIM28, effectively preventing the proteasomal degradation of CSDE1. As a consequence of this interaction, CSDE1 was enabled to coordinate oncogenic RNA regulons, which in turn activate the ERK pathway, a critical player in the prognosis of HCC [121].

Lastly, the lncRNA miR4458HG was discovered to influence HCC-cell proliferation, activate the glycolysis pathway, and promote tumor-associated macrophages’ polarization, highlighting its oncogenic role in HCC patients with high glucose metabolisms [122]. The lncRNAs regulated by m^6^A in HCC are summarized in Table 2.

In conclusion, these findings underscore the multi-faceted influence of m^6^A modification in the regulation of lncRNA functions, thereby controlling the progression of HCC. The m^6^A–lncRNA axis provides a connection between RNA modification and the complex networks of non-coding RNAs, potentially offering some insights into HCC pathogenesis, suggesting possibilities for new therapeutic approaches.

## 6. LncRNAs as Serum Biomarkers in Liver Cancer

Beylerli et al. highlighted that lncRNAs are secreted by tumor cells into human biological fluids, forming stable circulating lncRNAs resistant to RNA degradation. Aberrant expression of these lncRNAs has been observed in cancer patients [126]. Thus, for HCC diagnosis and prognosis, lncRNAs are increasingly being recognized as a potent alternative to traditional biomarkers such as alpha-fetoprotein (AFP). The efficacy of AFP as an early detector for HCC has been debated due to concerns regarding its sensitivity and specificity [127,128,129,130,131,132]. LncRNAs offer enhanced sensitivity and specificity, potentially addressing the limitations posed by traditional markers such as AFP [133,134,135]. Consequently, there is a growing demand for new diagnostic markers that could replace AFP, and lncRNAs could serve as one such alternative. Therefore, the expression levels of these lncRNAs could serve as key indicators of disease progression and prognosis.

Notably, lncRNAs such as MVIH, X91348, and HOTTIP have shown potential as prognostic markers in HCC [136,137,138]. The high expression of MVIH, associated with microvascular invasion, is a known independent risk factor for recurrence-free survival and overall survival in HCC patients [136]. Similarly, the low expression of lncRNA X91348 in HCC patients relative to healthy individuals was associated with increased overall survival [137]. Moreover, elevated HOTTIP levels were linked to increased tumor recurrence and decreased survival rates in HCC patients following liver transplantation. Conversely, a decrease in HOTTIP expression correlated with more favorable patient outcomes. Hence, HOTTIP could serve as a significant prognostic marker and potential therapeutic target for HCC [138].

With the progression towards minimally invasive and non-invasive diagnostic techniques, circulating lncRNAs in serum are being extensively studied. For instance, high serum levels of lncRNA-ATB were associated with overall survival, progression-free survival, tumor size, TNM stage, C-reactive protein levels, T stage, and portal vein thrombosis, highlighting their potential as serum biomarkers in HCC patients [139].

Exosomal lncRNAs present multiple benefits when considered as biomarkers. Protected from degradation by RNases within exosomes, these lncRNAs remained stable and detectable in various body fluids, making them a potential non-invasive diagnostic tool [135,140,141,142,143,144]. Moreover, given the tissue- or disease-specific nature of many lncRNAs, the detection of specific exosomal lncRNAs might indicate distinct cancer types. For instance, a signature composed of two lncRNAs, PVT1 and uc002mbe.2, demonstrated satisfactory sensitivity and specificity values for distinguishing liver cancer patients from healthy individuals, thus underscoring their potential as specific biomarkers for HCC [145]. Moreover, the lncRNA LINC00853 not only possessed potential diagnostic value, but it also showed prognostic relevance in HCC. Importantly, increased expression of LINC00853 is associated with lower survival rates in patients with stage II HCC according to the modified Union for International Cancer Control (mUICC II). This underlines the potential of LINC00853 as a liver-cancer-specific marker, providing an avenue for both disease identification and assessment of its progression [134].

Furthermore, several studies identified lncRNAs, such as UCA1 and WRAP53, as promising biomarkers in HCC diagnosis when used in conjunction with AFP [146,147]. Another group also identified LINC00152, RP11-160H22.5, and XLOC014172 as new biomarkers for HCC. These lncRNAs, in combination with the conventional marker AFP, were found to improve the diagnostic accuracy for HCC, indicating their potential for enhancing HCC diagnosis [148]. 

Within the context of chemotherapy resistance, certain lncRNAs, such as CAHM, were identified as key predictive markers. Utilizing machine learning algorithms, CAHM was characterized as a central lncRNA, with elevated expression in sorafenib-resistant cell lines, highlighting its prospective role as a biomarker for chemotherapy resistance [149]. The names and expression tendencies of lncRNAs with diagnostic potential for HCC are summarized in Table 3.

In summary, lncRNAs have emerged as promising biomarkers for HCC, with their roles in diagnostic and prognostic precision medicine becoming evident. Nonetheless, the literature lacks comparative studies between established biomarkers like PIVKA-II and new lncRNA biomarkers [160]. To validate lncRNAs’ clinical significance, extensive multicentric studies are essential. Additionally, standardizing methodologies for detecting circulating lncRNAs is vital to ensure consistent results. Despite challenges, exploring lncRNAs as HCC biomarkers promises to enhance diagnosis, prognosis, and targeted treatments for this malignancy.

## 7. Summary and Future Perspectives 

The discovery and subsequent study of lncRNAs significantly reshaped our understanding of genomic regulation. These non-coding transcripts, although initially dismissed as incidental transcriptional byproducts, have since been revealed as vital players in gene expression regulation and various biological processes [161,162]. This is particularly true for HCC, where lncRNA dysregulation is closely linked to disease pathogenesis [31].

Fortunately, the environment and numerous techniques for lncRNA research are steadily improving, and there is a wealth of web-based tools and publicly available data to facilitate the study of lncRNAs. These resources have expanded our ability to explore the multifaceted roles of lncRNAs and the mechanisms underlying their regulation (Table 4).

Such advancements in bioinformatics technology have further facilitated our ability to uncover the roles of lncRNAs in HCC, from influencing disease pathogenesis to acting as potential diagnostic markers. One such example is the exploration of upstream regulators like the m^6^A modification, unveiling lncRNA regulation and adding complexity to our understanding of their role in HCC. While current research predominantly revolves around m^6^A and its relationship with lncRNA, there are various RNA modifications similar to m^6^A, such as 5-methylcytosine (m^5^C), N^7^-methylguanosine (m^7^G), and 3-methylcytidine (m^3^C) [177]. The roles and biological functions of these diverse RNA modifications are not yet well-understood. Given this, they present promising research topics in relation to cancer etiology.

In future studies, research on lncRNAs holds promising potential to open new avenues for therapeutic intervention. Despite initial uncertainties and controversies, the role of lncRNAs in cellular function regulation and their implications in HCC have underscored their importance in biomedical research. Subsequent research endeavors should persist in elucidating the complexities of lncRNA function and dysregulation, deepening our comprehension of HCC, and establishing the foundation for novel diagnostic and therapeutic approaches. The challenge will be to translate this expanding knowledge into clinical applications, advancing lncRNA research from the bench to the bedside.

## Figures and Tables

**Table 1:** LncRNAs with differential expression profiles and their roles in hepatocarcinogenesis.

Expression	lncRNA	Known Function	Reference
Upregulated	HULC	EMT, metastasis, apoptosis	[56,67,68]
	MALAT1	EMT, metastasis, apoptosis, cell-cycle arrest	[61,65]
	HOTAIR	EMT, metastasis, apoptosis, cell-cycle arrest	[34,35,36]
	DLEU2	Vascular invasion, lymphatic metastasis	[69]
	SNHG1	EMT, cell-cycle regulation, metastasis, apoptosis	[70]
	NEAT1	Ferroptosis, metastasis, proliferation, invasion, drug resistance	[47,51,53,54,71]
	TUG1	Metastasis, apoptosis	[72]
	CRNDE	Proliferation, migration, invasion	[73]
	KDM4A-AS1	EMT, metastasis	[74]
	UCA1	EMT, cell-cycle regulation, apoptosis	[75]
	ANRIL	Metastasis, apoptosis	[76,77]
	CASC15	EMT, metastasis, suppression of apoptosis	[78]
	ZFAS1	Metastasis	[79]
	CARLo-5	EMT	[80]
	LOC90784	Apoptosis, cell-cycle arrest	[81]
	H19	EMT, metastasis, suppression of apoptosis	[82,83,84]
	PCAT-14	cell-cycle arrest	[85]
	LINC02551	EMT, metastasis	[86]
	LINC01116	EMT, cell-cycle regulation, metastasis, immune-cell infiltration	[87]
	CCAT2	Proliferation, migration, invasion	[88]
	PVT1	Microvascular invasion, proliferation	[89,90]
Downregulated	DGCR5	Proliferation, migration, invasion	[91]
	MEG3	Inhibition of metastasis, angioinvasion and proliferation by cell-cycle regulation	[92,93,94]
	FENDRR	Apoptosis, Treg-mediated immune escape	[95,96]
	GAS5	Suppression of proliferation, drug resistance and M2 macrophage polarization	[97,98]
	RAB11B-AS1	Apoptosis	[99]

**Table 2:** m^6^A modified lncRNAs in HCC.

Expression	lncRNA	m^6^A Binding Partner	Reference
Upregulated	HULC	IGF2BP1	[123]
	LINC00958	METTL3	[118]
	LINC02362	-	[119]
	SNHG20		
	SNHG6		
	MIR4458HG	IGF2BP2	[122]
	LINC02551	ALKBH5	[86]
	MIR155HG	METTL14	[120]
	SLC7A11-AS1	METTL3	[124]
	ARHGAP5-AS1	METTL14 IGF2BP2	[121]
Downregulated	RAB11B-AS1	METTL16	[99]
	MEG3	METTL3	[94]
	AC115619	WTAP	[125]

**Table 3:** Potential lncRNAs as diagnostic biomarkers for HCC detection.

Expression	lncRNA	Blood Detection	Reference
Upregulated	MVIH	Yes	[136]
	HOTTIP	Yes	[135,138]
	ATB	Yes	[139]
	DLEU2	Yes	[135]
	MALAT1	Yes	
	SNHG1	Yes	
	PVT1	Yes	[145]
	uc002mbe.2	Yes	
	LINC00853	Yes	[134]
	UCA1	Yes	[146,147]
	WRAP53	Yes	[147]
	LINC00152	Yes	[148]
	RP11-160H22.5	Yes	
	XLOC014172	Yes	
	DANCR	-	[150]
	LINC00978	Yes	[151]
	LncDQ	Yes	[152]
	SPRY4-IT1	-	[153]
	UBE2CP3	Yes	[133]
	LINC01225	Yes	[154]
	H19	Yes	[155]
	uc003wbd	Yes	[156]
	CAHM	-	[149]
	FAM72D-3	Yes	[157]
	EPC1-4	Yes	
Downregulated	JPX	-	[158]
	XIST	-	
	DGCR5	-	[159]
	X91348	Yes	[137]

**Table 4:** Web-based tools for comprehensive lncRNA research and analysis.

Name	Website	Description	Reference
RNACentral	https://rnacentral.org (accessed on 10 September 2023)	A public platform offering access to a wide collection of non-coding RNA sequences from various organisms and RNA types	[163]
LncBase	https://diana.e-ce.uth.gr/lncbasev3 (accessed on 10 September 2023)	A database cataloging 500,000 verified miRNA–lncRNA interactions across 243 cell types	[164]
RNAfold	http://rna.tbi.univie.ac.at/cgi-bin/RNAWebSuite/RNAfold.cgi (accessed on 10 September 2023)	A tool used for predicting the secondary structure of RNA sequences	[165]
LncSEA	http://bio.liclab.net/LncSEA/index.php (accessed on 10 September 2023)	A platform for lncRNA-related sets and enrichment analysis	[166]
LncExpDB	https://ngdc.cncb.ac.cn/lncexpdb (accessed on 10 September 2023)	Expression database of human lncRNAs	[167]
lncRNAKB	https://bio.tools/lncrnakb (accessed on 10 September 2023)	A knowledgebase of tissue-specific functional annotation and trait association of lncRNA	[168]
LNCipedia	http://www.lncipedia.org (accessed on 10 September 2023)	A database for annotated human lncRNA transcript sequences and structures	[169]
dbEssLnc	https://esslnc.pufengdu.org (accessed on 10 September 2023)	A manually curated database of human and mouse essential lncRNA genes	[170]
LncTar	http://www.cuilab.cn/lnctar (accessed on 10 September 2023)	A tool for predicting the RNA targets of lncRNAs.	[171]
TANRIC	https://bioinformatics.mdanderson.org/public-software/tanric (accessed on 10 September 2023)	TANRIC webapp provides analysis of lncRNA in cancer, highlighting potential therapeutic targets and biomarkers	[172]
LncBook	https://ngdc.cncb.ac.cn/lncbook (accessed on 10 September 2023)	A comprehensive database of human lncRNAs, offering annotations for understanding their roles in diseases and biological contexts.	[173]
lncATLAS	https://lncatlas.crg.eu (accessed on 10 September 2023)	A database showing subcellular locations of GENCODE-annotated lncRNAs, using RCI values	[174]
RNAInter	http://www.rnainter.org (accessed on 10 September 2023)	A database with a scoring system to rate the confidence of RNA-associated interactions based on experimental evidence and tissue/cell types	[175]
ENCORI	https://rnasysu.com/encori (accessed on 10 September 2023)	A platform for studying RNA interactions, integrating diverse data and enabling pan-cancer analysis	[176]

## Data Availability

Data sharing not applicable.
